# Analysis of spontaneous reports of suspected adverse reactions after vaccination against COVID-19 in Slovakia

**DOI:** 10.3389/fphar.2023.1097890

**Published:** 2023-01-16

**Authors:** Monika Lassanova, Stefan Lassan, Silvia Liskova, Tomas Tesar, Monika Cicova

**Affiliations:** ^1^ Institute of Pharmacology and Clinical Pharmacology, Faculty of Medicine, Comenius University, Bratislava, Slovakia; ^2^ Department of Pneumology, Phthisiology and Functional Diagnostics, Slovak Medical University and Bratislava University Hospital, Bratislava, Slovakia; ^3^ Department of Organisation and Management of Pharmacy, Faculty of Pharmacy, Comenius University, Bratislava, Slovakia; ^4^ State Institute for Drug Control, Pharmacovigilance and Clinical Trial Section, Bratislava, Slovakia

**Keywords:** SARS-CoV-2 virus, COVID-vaccines, Spikevax, Comirnaty, Vaxzevria, spontaneous reports of adverse reactions, adverse reaction (ADR)

## Abstract

**Introduction:** The COVID-19 pandemic has resulted in more than 6.5 million deaths worldwide yet. Vaccination against the SARS-CoV-2 virus is a reliable way out of the pandemic, however, vaccination rate reaches only 58% in the Slovak Republic. Concerns about the adverse reactions of vaccines are one of the reasons for the low vaccination rate.

**Objective:** The aim of our analysis was to review reported suspicions of adverse reactions (ARs) of registered COVID-19 vaccines (Comirnaty, Vaxzevria, Spikevax), which State Institute for Drug Control received from healthcare professionals and patients in the period from 1 January 2021 to 31 May 2021.

**Methods:** Data were collected from the State Institute for Drug Control database, a retrospective analysis was carried out focusing on trends in the number of all reports of suspicions of adverse reactions sent to the State Institute for Drug Control during the previously mentioned period. We analysed the Retrieved data were analysed with the usage of descriptive statistics and comparison to historical data on drug adverse reactions in Slovakia was performed.

**Results**: During the evaluation period, 5,763 reported suspicions of adverse reactions were analysed, overall, there was a significant (*p* < 0.0001) increase in the number of reported adverse reactions fivefold. 93% of ARs (*n* = 5,346) were reported for COVID-19 vaccines. In comparison of the extentof all adverse reactions, there is clearly a statistically significant difference between all types of vaccines administered at that time (*p* ≤ 0.0001). No statistically significant difference (*p* ≤ 0.238) was identified between Spikevax and Comirnaty in the proportion of serious adverse reactions. However, a significantly higher (*p* ≤ 0.00001) proportion of reported suspicions of serious adverse reactions was observed after the administration of Vaxzevria.

**Conclusion:** This is the first analysis conducted in Slovakia aimed to reported adverse reactions in relation to the administration of COVID-19 vaccines. The rate of spontaneously reported suspected adverse reactions has been insufficient in the past for a long time; during the period from January to May 2021 the reporting rate increased due active calls for adverse reactions reporting. In concordance with European data, Vaxzevria had a significantly higher ratio of reported suspicions of serious adverse reactions.

## 1 Introduction

The Coronavirus disease 2019 (COVID-19) pandemic may be considered the largest public health challenge of the present millennium. It has resulted in over 6.5 million deaths worldwide as of the end of November 2022 and is associated with devastating social and economic impacts as well (World Health Organisation 2022). An urgent need quickly arose to stop the global crisis and mitigate its consequences. The population-wide implementation of COVID-19 vaccination represents a crucial tool for limiting disease transmission and a means of achieving so-called “herd immunity” (Mohammed et al., 2022). Therefore, due to enormous efforts, the development of vaccines against the SARS-CoV-2 virus followed rapidly and resulted in the registration of vaccines against the SARS-CoV-2. As of 20 December 2021, five vaccines against the SARS-CoV-2 are conditionally registered in the European Union (EU), namely: Comirnaty (Pfizer/BioNTech), Spikevax (Moderna), Vaxzevria (AstraZeneca) JCOVDEN (Janssen—Johnson & Johnson) and Nuvaxovid (Novavax).

Following the urgency of anti-pandemic measures, the European Medicines Agency (EMA) introduced several procedures, including a rolling review (a continuous assessment process), with the aim of accelerating the registration of safe, effective and high-quality vaccines and therapeutics against COVID-19. Even for these conditionally registered preparations against COVID-19, it is mandatory that during the entire period, their benefits for the patients’ health must exceed the potential risks of damage to health. Close monitoring and studying of complications associated with COVID-19 vaccines in both adult and paediatric populations is urgently required (Bilotta et al., 2022).

From a public health viewpoint, several important issues are still present and relevant in COVID-19 vaccines. They not only relate to the duration of vaccine efficacy and protection, but also to safety (Sessa et al., 2021). Measures such as clarifying vaccine safety and effectiveness are essential to reduce vaccine hesitancy in the general population (Pomara et al., 2021a). Allegations that vaccines/vaccination cause adverse events must be dealt with rapidly and effectively. Vaccine-associated adverse reactions (ARs) and error-related immunisation events may affect healthy individuals and should be promptly identified for further response. In order to satisfy the global interest in developing a scientific methodology for vaccine pharmacovigilance systems, the World Health Organisation published a revised user manual for causality assessment of an adverse event following immunisation (AEFI) (World Health Organisation, 2018).

Concerns about the safety of vaccines against the SARS-CoV-2 virus appear to be the most significant limiting factor of the low vaccination rate in Slovakia (Národné centrum zdravotníckych informácií, 2021; Ulbrichtova et al., 2021). Reports of suspected ARs of medicines or vaccines are a part of pharmacovigilance. The collection and analysis of this data make it possible to monitor the safety of medicines and vaccines after their launch on the market and to obtain new safety information. The level of spontaneous reports of suspected adverse reactions has been low for a long time in the Slovak Republic, which is also confirmed by the Annual Reports of State Institute for Drug Control (SIDC) (Varga et al., 2020; Laššánová and Čičová, 2021; ŠÚKL, 2022). During the vaccination against the COVID-19 disease, those vaccinated in Slovakia were actively encouraged to report suspicions to the National Health Service, which is why there was a thirteen-fold increase in the number of reports compared to previous years (Table 1).

**TABLE 1:** Numbers of suspected ARs reported to the State Institute for Drug Control during the period from 1 January 2006 to 31 May 2021 in the Slovak Republic.

Year	ARs-SIDC in total (n)	ARs of medication (n)	ARs of vaccines (n)	Serious ARs of vaccines (n)	Non-serious ARs of vaccines (n)
2006	879	571	308	35	273
2007	1,206	646	560	42	518
2008	845	687	158	31	127
2009	1,037	906	131	35	96
2010	1,001	919	82	28	54
2011	1,030	757	273	72	201
2012	967	764	203	54	149
2013	1,038	880	158	60	98
2014	1,059	972	87	19	68
2015	1,071	966	105	41	64
2016	1,470	1,349	121	66	55
2017	1,723	1,614	109	63	46
2018	956	889	67	20	47
2019	1,128	1,053	75	15	60
2020	821	756	65	6	59
01–05/2021	5,763*	372**	5,391	393	4,998
**(N)**	**21,994**	**14,101**	**7,893**	**980**	**6,913**

The bold values represent total figures.

*p < 0.0001, **p ≤ 0.589.

The aim of our analysis of reported suspicions of ARs in a temporary relationship with vaccination against the SARS-CoV-2 virus was to clarify the aspects of Ars, with a further goal of advancing our knowledge for supporting decisions or suggesting changes in policies at the national level in the Slovak Republic. Moreover, our pilot work intends to improve general awareness of vaccine safety.

## 2 Objective

The aim of the presented pilot work was an analysis of reported suspicions of ARs—their frequency, severity and nature—obtained from the database of the SIDC for conditionally registered and used vaccines against COVID-19 from 1 January 2021–31 May 2021.

The SIDC is the national medicine agency of the Slovak Republic responsible for the receiving, collecting, recording and processing of data on the safety of drugs and vaccines. It also participates in the assessment of the risks of using pharmacological treatment. Data from the SIDC represent a relevant basis to objectively assess the risks of any treatment as well as vaccines in clinical practice in the Slovak Republic.

## 3 Materials and methods

### 3.1 Methodology

Based on data collected from the SIDC database, we evaluated in a retrospective analysis the trends in the number of all reports of suspicions of AR sent to the SIDC for the period 1 January 2006 to 31 May 2021. We focused on data on the reports of suspicions of ARs which were sent to the Clinical Trials and Pharmacovigilance Section of the SIDC by health professionals and vaccinated people after vaccination with conditionally registered vaccines for the prevention of the COVID-19. All vaccines licenced in Slovakia as of 31 May 2021, namely Comirnaty (Pfizer/BioNTech), Spikevax (Moderna), Vaxzevria (AstraZeneca) and JCOVDEN (Janssen—Johnson & Johnson), are included in the data set. Neither Nuvaxovid (Novavax) nor Sputnik (Gamaleya Institute) are part of the analysis, as they were not yet marketed in the Slovak Republic as of 31 May 2021. The reports of the marketing authorisation holders were not included in the analysis, because they are obliged to report suspicions of acute respiratory infections directly to the European database—EudraVigilance—within a time frame of 15–90 days, depending on the severity of the AR (European Medicines Agency, 2018).

### 3.2 Eligibility, inclusion, and exclusion criteria

Only reports of suspected ARs sent to the SIDC in temporary relationship with vaccination for the prevention of COVID-19, in which the full/exact name of the vaccine, active substance or manufacturer was given, were statistically evaluated. Reports of suspected ARs to the SIDC without these data were not included in the analysis (26). Due to this, the numbers may differ slightly from the total number of reported ARs of vaccines against COVID-19 shown in Table 1. During the analysed period from 1 January 2006 to 31 May 2021, 2,619,950 individual doses of the three vaccines against COVID-19—Comirnaty, Vaxzevria and Spikevax—were administered in the Slovak Republic. The data on which we based our calculations were obtained from the reference source of the National Health Information Centre (Národné centrum zdravotníckych informácií, 2021) (Table 2).

**TABLE 2:** Reported suspected ARs after the administration of all vaccines for the prevention of COVID-19 from 1 January 2021 to 31 May 2021.

Vaccine	Administered doses (n)	ARs (n; %)	Non-serious ARs (n)	Serious ARs (n)
Comirnaty (Pfizer/BioNTec)	1,752,866	2,232 (0.127%)	2,058	174
Spikevax (Moderna)	262,546	455 (0.173%)	423	32
Vaxzevria (AstraZeneca)	604,538	2,633 (0.435%)	2,452	181
All COVID-19 vaccines	**2,619,950**	**5,320 (0.203%)**	**4,933** (92.7%)	**387** (7.3%)

The bold values represent total figures.

### 3.3 Terminology

For terminology, we used generally accepted nomenclature and definitions. ARs are defined as harmful and unwanted reactions that occur after the use of a drug or vaccine at a therapeutic dose, where there is at least a reasonable possibility of a causal relationship between the drug or vaccine and the reaction (Eur-lex, 2001; European Medicines Agency, 2017). In the evaluation of ARs, a serious AR of a human medicine is one that causes death, threatens a patient’s life, requires institutional healthcare or the prolonging of the institutional healthcare, causes disability or an incapacity to work, the patient’s invalidity or is manifested by a congenital anomaly or malformation (Národná rada SR, 2011). According to the EMA’s Good Pharmacovigilance Practice Recommendations (European Medicines Agency, 2017), one of the severity criteria is also an “otherwise medically significant condition”.

### 3.4 Statistical analysis

We analysed the extracted data using descriptive statistics and displayed them graphically using Excel (Microsoft Office 2016). We statistically analysed the data of suspicion of ARs from the SIDC database and determined the 95% confidence interval using the Clopper and Pearson exact method. We tested the determined null and alternative hypotheses with a binominal test. For the statistical evaluation of the data, we used the program R version 3.6.3 (R Foundation for Statistical Computing, Vienna, Austria).

## 4 Results

**FIGURE 1:**
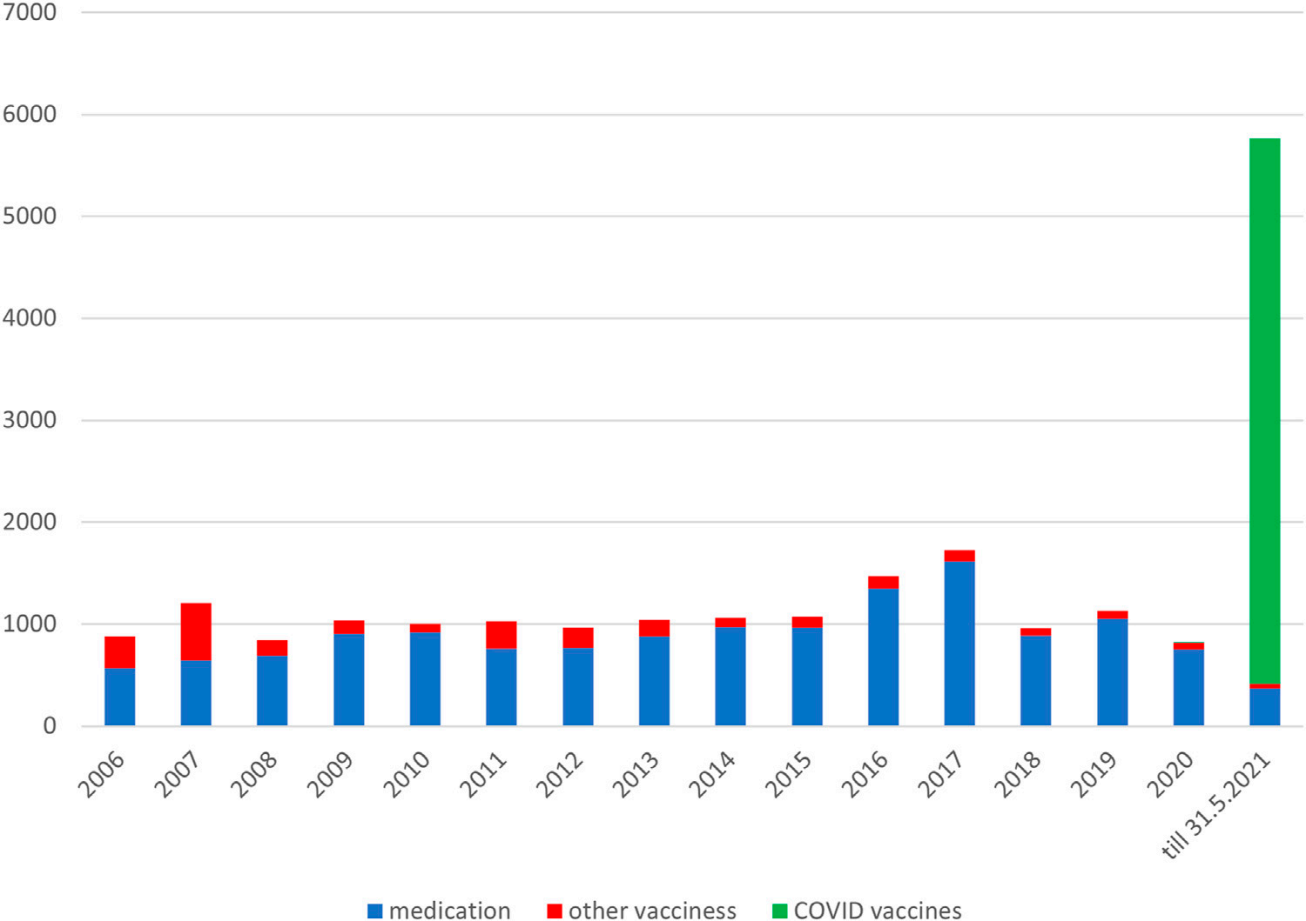
Reported suspected ARs from 1 January 2006 to 31 May 2021.

**FIGURE 2:**
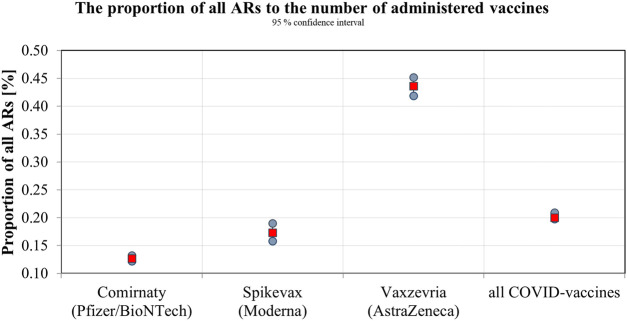
The proportion of all ARs to the number of administered vaccines.

**FIGURE 3:**
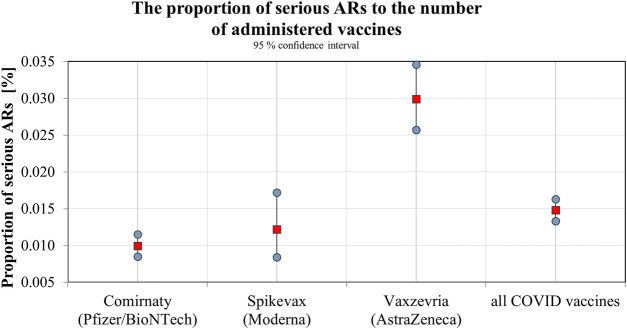
The proportion of serious ARs to the number of administered vaccines.

The SIDC registered a total of 21,994 reported suspicions of ARs from 1 January 2006 to 31 May 2021 (Table 1; Figure 1). Out of this total, 7,893 (36%) represent suspected cases of ARs after administration of vaccines and 14,101 (64%) reported cases of ARs after medication. In the pilot analysis of spontaneous reports of ARs at the SIDC in connection with vaccination against COVID-19, we focused more closely on the period from 1 January 2021 to 31 May 2021, in which 5,763 of all suspected ARs (medications and vaccines) were reported to the SIDC. After extrapolation to 12 months, the period January to May 2021 showed a significant increase in the number of reported suspicions of ARs to the SIDC. In the first 5 months of 2021, the average annual number of reports of suspected ARs to the SIDC (for the period 2006–2020) was enhanced five-fold (*p* < 0.0001). At the same time, there was no statistically significant change in the total number of reported suspected ARs at the SIDC for medicines. There was no significant difference in the number of reports, because the value extrapolated from 5 to 12 months is 893 and the long-term average from the years 2006–2020 is 915 (*p* ≤ 0.589). Out of the total number of 5,763 reports sent to the SIDC during the period January to May 2021, 372 (6%) reports of ARs were for medicines, 45 (1%) were reports of ARs for other vaccines (excluding vaccines against COVID-19), and 5,346 (93%) were for vaccines used in the Slovak Republic to prevent COVID-19. The proportion of all analyzed 5,320 ARs in the total number of administered vaccines is shown in Figure 2. When comparing the representation of all ARs, there is clearly a statistically significant difference between all types of vaccines administered at that time (*p* ≤ 0.0001). 387 (7.3%) of 5,320 reports for all vaccines met the severity criterion in reports (Table 2). The proportion of serious ARs in the number of vaccines administered is illustrated in Figure 3. No statistically significant difference (*p* ≤ 0.238) was identified between Spikevax and Comirnaty in the proportion of serious ARs. However, a significantly higher (*p* ≤ 0.00001) proportion of reported suspicions of serious ARs was observed with Vaxzevria. The most frequently reported suspicions of serious ARs following administration od COVID-19 vaccines are summarised in Table 3 **.** The SIDC informs the public only about closed cases of death in causal relationship with the vaccination. As of 31 May 2021, a total of three deaths related to vaccination were recorded in the Slovak Republic, of which a causal relationship was established as possible in two cases (after administration of Comirnaty and Spikevax vaccines, respectively) and as probable in one case (after administration of the Vaxzevria vaccine). An forensic autopsy was performed in all three cases, as post-mortem investigation plays pivotal role in the assessment of the causality relationship (World Health Organisation, 2018; Pomara et al., 2021a). In the case of both deaths with possible causal relationship to the prior vaccination, the affected persons were frail elderly patients (aged 79 and 79 years, respectively) suffering from several chronic diseases. With reference to the autopsy reports, the cause of death was related to chronic underlying diseases to which mild adverse effects (fever, weakness) may have contributed. The latter case of death with proble causal relationship applies to a 47-years-old woman with genetic predisposition to a thrombophilic state according to previous examination history. The autopsy revealed cerebral venous thrombosis. In other closed cases, the connection was excluded, e.g., based on information from the autopsy report of the Healthcare Surveillance Authority. Even if the relation was not confirmed, but the death was reported to the SIDC, the report is sent together with its description to the European database of ARs (EudraVigilance) (Štátny ústav pre kontrolu liečiv, 2021).

**TABLE 3:** The most frequently reported suspicions of serious adverse reactions after the administration of vaccines for the prevention of COVID-19 until 31 May 2021.

Vaccine	Reported suspected serious adverse reactions
Comirnaty (Pfizer/BioNTech)	Persistent increase in blood pressure (mostly in patients treated for hypertension), pre-collapse state, collapse, thrombosis, allergic reactions (1 anaphylactic reaction), facial nerve paresis, vaccination failure, phlebothrombosis, thrombosis, pulmonary embolism
Spikevax (Moderna)	Temporary paralysis, persistent increase in blood pressure, thrombosis, loss of consciousness
Vaxzevria (AstraZeneca)	Short-term loss of consciousness, thrombosis, and phlebothrombosis of the lower extremities, persistent increase in blood pressure, paresis of the facial nerve, epileptic seizure in patients treated for epilepsy, anaphylactic reaction, erysipelas, hyperpyrexia, phlebitis of the lower extremities, pulmonary embolism, sudden cerebrovascular accident

## 5 Discussion

In the presented study, we performed the first analysis in Slovakia focused on the occurrence of ARs after the administration of COVID-19 vaccines. If we focused on the average annual number of reports of suspected ARs to drugs and vaccines, it can be concluded that in the period January to May 2021, there was a significant increase in the number of reported suspicions at the SIDC, roughly five-times the annual average for the years 2006–2020 (*p* < 0.0001). At the same time, there was no significant change in the number of reports of suspicions to the SIDC for medicines (*p* ≤ 0.589). Despite this increase in reports, the awareness of the need to inform the patient about the safety of the treatment, but also about the need to report suspicions of ARs, is lower in Slovakia compared to other countries, which was also confirmed by the study of Varga et al. (2020). The analysis proved that there was no statistical difference in the occurrence of ARs between Comirnaty and Spikevax vaccines, in contrast to the Vaxzevria vaccine, where a significantly higher proportion of reported suspicions of ARs were recorded.

Thus, a significant statistical increase was caused by reports of suspicions at the SIDC in connection with vaccination for the prevention of COVID-19 (they account for 93% of the total number of reports for the period January to May 2021). This confirmed the impact of media coverage on the number of reports, especially from the lay public (in our data set, the lay public filed almost 64% of reports), similar to that published in studies by Martin et al. in connection with the use of paroxetine, or Postman et al. in connection with the use of oral hormonal contraception (Martin et al., 2006; Postma and Donyai, 2021). Other EU and non-EU countries have also observed an increase in reports of suspected ARs. The EMA recorded 3.5 million reports in 2021—the largest number of reports recorded in 1 year to date—with 1.68 million (48%) reports related to the use of vaccines to prevent COVID-19. This proportion is significantly lower than in our group (93%) (European Medicines Agency, 2021a). A similar increase in the number of reports of acute respiratory infections following COVID-19 vaccines is also recorded by the WHO international monitoring centre in Uppsala (Uppsala Monitoring Centre), where from January to June 2021 over 1 million reports were recorded, making vaccines against COVID-19 the most reported component in the VigiBase database (World Health Organisation, 2021).

In a more detailed retrospective analysis, we focused on the frequency, seriousness and nature of spontaneous reports of suspicions of ARs at the SIDC in connection with vaccination against COVID-19. During the analysed period, three vaccines were used, two of which belong to the group of mRNA vaccines (Comirnaty and Spikevax) and the third belongs to the group of so-called vector vaccines (Vaxzevria). During the first 5 months, 2,619,950 doses (Národné centrum zdravotníckych informácií, 2021) were administered in the Slovak Republic, while the representation of individual vaccines was as follows: 66.91% Comirnaty; 10.02% Spikevax, and 23.07% Vaxzevria. There were 5,320 reports of suspected ARs of vaccination recorded during the first 5 months of 2021; however, the incidence rate of the seriousness of ARs was not related to the number of administered vaccines. We observed a statistically significant difference between all vaccines in terms of the rate of occurrence of all ARs (*p* < 0.001, p_max_ ≤ 2.7 × 10^−10^). Serious ARs accounted for 7.3% of the total number; in terms of their rate of occurrence, this is in accordance with an American study, where serious ARs accounted for 6.6% of the total, while in their study only mRNA vaccines were analysed (Rosenblum et al., 2022). We identified a statistically significant difference of serious ARs only after active immunization with the Vaxzevria vaccine (*p* ≤ 0.00001), and on the contrary, we did not identify them in their occurrence after the administration of the Spikevax and Comirnaty vaccines. As of 31 May 2021, a total of three deaths related to vaccination were recorded in the Slovak Republic, of which a causal relationship was established as possible in two cases (after administration of Comirnaty and Spikevax vaccines, respectively) and as probable in one case (after administration of the Vaxzevria vaccine). All three fatal cases were further investigated by the SIDC and autopsies were performed. In accordance with generally accepted authorities, we agree with the recommendation that autopsy should be the rule in the causality assessment of fatal cases occurring in the time context of the vaccination against SARS-CoV-2 virus (Sessa et al., 2021). In this regard, one case of fatal cerebral venous thrombosis in a female patient with a previous history of a predisposition to thrombophilic complications was reported during the monitored period. The causal association between the event and prior vaccination with a adenoviral-based vaccine (Vaxzevria) was determined to be probable. Although rare, vaccine-induced immune thrombotic thrombocytopenia and related events (e.g., cerebral venous thrombosis) after the receipt of the adenoviral-vector vaccines may seldom occur even in otherwise healthy young adults (Pomara et al., 2021b; Greinacher et al., 2021; Schultz et al., 2021; Li et al., 2022). Similarly, a British study documents that fatal adverse reactions after vaccines against COVID-19 are rare (Ferner et al., 2022). The European Medicines Agency (EMA) is closely monitoring the safety of the COVID-19 vaccines and has concluded that the vast majority of known ARs of the COVID-19 vaccines are non-serious and not permanent (European Medicines Agency, 2021b).

Despite our goal to provide a comprehensive overview of safety data in the Slovak Republic for the studied period, our pilot analysis still has some important limitations. Above all, the absence of several data represents a setback worthy of mention. The sum of available data from reports of suspected ARs from the SIDC database may differ from the real number of manifested ARs in the Slovak Republic mainly for the following reasons: the low rate of reporting of suspected ARs in clinical practice, primarily by healthcare professionals; incompleteness of clinical data in reports (gender, accurate identification vaccines); missing exact data on comorbidities of the enrolled cases, with the exception of fatal ARs (where they were actively traced); failure to include invalid reports of ARs and reports sent by Marketing Authorisation Holders in the Slovak Republic/EU; incorrect evaluation of the causality of ARs by patients or healthcare professionals; stimulated collection of ARs or even financial motivation of patients due to requests for financial compensation for ARs. Exact data on the manifestation of ARs in clinical practice can only be obtained in a strictly controlled set of patients (a clinical trial).

Moreover, after close evaluation of more relevant scientific data, it will be possible to understand the risk/benefit ratio in all relevant subsets, including the paediatric population (Bilotta et al., 2022). However, clinical trials are not designed to detect very rare adverse events—this requires post-authorisation monitoring. Beyond the scope of data processing from pharmacovigilance, the application of a practical workflow to causality assessment of the temporary relationship between AEFI and COVID-19 vaccination is fully advocated. The proposed procedures are based on the mandatory four steps for each AEFI case. Following this algorithm, it would be possible to classify the AEFI. In particular, four categories have been proposed depending on the degree of verified causality (Pomara et al., 2021a).

Finally, the available evidence from our pilot analysis as well as from international studies and meta-analyses suggests that vaccines against COVID-19 have an acceptable short-term safety profile. At the same time, further studies and long-term follow-up at the population level are recommended to further define the safety, especially long-term, and the profile of vaccines against COVID-19 (European Medicines Agency, 2021b; Wu et al., 2021; Ferner et al., 2022; Rosenblum et al., 2022). The main reason rests in fact that the safety profile of the relatively recently introduced vaccines against SARS-CoV-2 virus still needs to be clarified in a changing environment of a growing target population with a variety of characteristics potentially influencing the outcome.

## 6 Conclusion

The vaccination against the SARS-CoV-2 virus could be considered a crucial intervention with great importance for public health. However, vaccine-related adverse reactions should not be exempt from future remediation. Reporting suspected ARs is the basis of the pharmacovigilance system. The goal of pharmacovigilance is the prevention of health damage from adverse reactions in humans and the promotion of safe and effective use of medicines and vaccines. Reports of suspected ARs contribute to better monitoring of ARs in clinical practice and help to obtain new information on the safety profile of a drug. In the set we analysed, we noted a significant, five-fold, increase in reports of suspicions of ARs that was due to induced reports of vaccines against COVID-19, primarily from the lay public. This is related to the active system of induction of reports after COVID-19 vaccines. In the Slovak Republic a vaccinated person received an SIDC form for reporting ARs directly at the clinic where the vaccination process took place, which had to be filled out and brought to the administration of the second dose of the vaccine. The media, which repeatedly and strongly reported on the problems and the ARs after the vaccines, also played a role, and the effort to obtain financial compensation for ARs may also have played a certain role. Among all the vaccines administered at that time, there is a significant difference in the proportion of all ARs. Among reports, 7.3% of 5,320 reports for all vaccines could be classified as serious. As of 31 May 2021, a total of three deaths related to vaccination were recorded in the Slovak Republic, of which the causal relationship was established as possible in two cases (after administration of Comirnaty and Spikevax vaccines respectively) and as probable in one case (after administration of the Vaxzevria vaccine). Although rare, the observed risks after adenovirus based vaccines should be considered when prescribing vaccines to individuals with different risk profiles in relation to potential AEFIs. Safety data from more than 2,619,950 doses of vaccines against COVID-19 (Comirnaty, Spikevax, Vaxzevria) administered during the first period from 1 January 2021 to 31 May 2021 of the Slovak vaccination program show that the more than 90% of reported ARs were non-serious, while the most frequently reported serious ARs were after the Vaxzevria vaccine. Based on our pilot analysis using local data, we can encourage the efforts aimed at raising awareness of generally favourable vaccination safety. This is particularly important in countries where vaccination coverage falls short of expectation, and the public’s attitude towards this public health intervention is insufficient. In order to translate these efforts into clinical practice, a basic framework applicable to the causality assessment of AEFI that occur after COVID-19 vaccination was recently proposed and strongly recommended. Causality evaluation of AEFIs is not only crucial to counteract current vaccine hesitancy and suspicion, but also for implementing an evidence-based vaccination policy (Pomara et al., 2021a).

## Data Availability

The original contributions presented in the study are included in the article/Supplementary Material, further inquiries can be directed to the corresponding authors.
